# Addressing Stillbirth: Exploring Economic Status, Government Strategies, and Gaps

**DOI:** 10.7759/cureus.76923

**Published:** 2025-01-04

**Authors:** S. Suganathan Soundararajan

**Affiliations:** 1 Community Medicine, PSG Medical College, Coimbatore, IND

**Keywords:** antenatal care, community, economic burden, economic status, financial incentives, global plans, global strategies, maternal and child health, public health, stillbirth

## Abstract

Stillbirth rates exhibit a strong correlation with a country’s economic level, suggesting the significant impact of socioeconomic factors on maternal and fetal health. Various risk factors for stillbirth have been identified in high-, low- and middle-income countries, but the widening economic inequity between different nations results in discrepant etiologies of stillbirth. The emotional impacts of stillbirth affect not only the birthing person, but also the reproductive partner, family, and community due to the enduring pain and grief associated with the loss. Herein, we review the existing government strategies and the limiting factors in implementing these plans across high-, middle-, and low-income countries and discuss possible ways to prevent stillbirth. Furthermore, we have mentioned various factors that are directly and indirectly associated with the national economic status and the possible ways to intervene in the rising stillbirth rate. We also discussed the additional psychological burden that arises from the financial crisis among people in low- and middle-income countries. While global strategies are being implemented to attain a single-digit stillbirth rate by 2030, underreporting of the incidence of stillbirth cases remains a major threat to the achievement of this goal. Standardizing guidelines for reporting and recording stillbirth cases will effectively help in achieving global targets. Financial incentive schemes along with the existing strategies will significantly reduce the stillbirth rates in nations.

## Introduction and background

Worldwide, the stillbirth rate declined from 21.4 per 1,000 total births in 2000 to 13.9 per 1,000 births in 2021. This reduction is around 35%. Despite this substantial progress, there were 1.9 million stillbirths in 2021 [1]. There are major disparities between nations and regions in reducing the stillbirth rates globally. Every 16 seconds, a stillbirth occurs somewhere in the world, among which 91% of intrapartum stillbirths occur in sub-Saharan Africa and South Asia [2].

Since stillbirth is multifaceted, the medical system alone cannot effectively alleviate the causes. The long-lasting impact of stillbirth places a heavy burden on parents, families, the community, society, policymakers, and public health professionals [3]. Particularly in low- and middle-income countries (LMICs), the economic and psychosocial cost has a major negative influence on the population [4]. The costs incurred by stillbirth begin during antenatal care and continue into the future for all those affected [5].

Proper prenatal and intrapartum care can avert nearly 40% of stillbirths [6]. According to the United Nations Inter-Agency Group for Child Mortality Estimation (UN IGME) report, stillbirths have not decreased as quickly as maternal and infant mortality since 2000 [7], and a further 19 million stillbirths will occur if the current scenario persists.

Although many studies have outlined the various predictors, causes, and determinants of stillbirth, the economic discrepancies between the nations and their impact on maternal health care are discussed in this review along with various existing strategies and plans to reduce preventable stillbirth. The present review includes studies that are concerned with the economic and social hardships in stillbirth across the countries.

## Review

Current scenario of stillbirth

Among the various maternal health complications, stillbirth is highly linked with economic status, emphasizing the major role of capital from conception to birth [8]; however, there is no monetary value placed on the loss of life. In a Lancet series titled “Ending preventable stillbirths,” the measured direct and indirect costs of stillbirth were higher than those for live births. The direct financial costs include expenditure on healthcare, medical care, investigations about the cause of death, and fetal surveillance after a stillbirth. Funeral and burial expenditures are added indirect financial burdens [4].

In Australia [9], England, and Wales [10], stillbirths cost more than live births. As with the stillbirth rates in the LMIC, the economic burdens are also underreported [11]. Studies have estimated that the direct expenses associated with each stillbirth ranged between $6,934 and $9,220. Indirect costs were found to constitute approximately 97% of the total expenses. However, the inclusion of intangible costs remained largely unexplored, leaving this aspect insufficiently studied [12].

The risk factors associated with stillbirth in HIC include maternal age and weight, lifestyle, substance use, and physical inactivity [13], while low antenatal care, lack of health care system, socioeconomic status, infections, maternal malnutrition, and history of stillbirth are the major risk factors of stillbirth in LMIC [14]. Although low socioeconomic condition was a major financial cause of stillbirth in LMIC, the financial burdens in HIC were also high, despite the lower stillbirth rates, due to the prevention costs associated with education about smoking cessation among women [15].

Additionally, the stillbirth burden is not limited to only financial; the major psychological impacts of stillbirth on pregnant people and their families include pain, grief, depression, anxiety, guilt, and post-traumatic stress disorder [5,16]. There is an additional burden on people who experience stillbirth in LMIC; including constant stigmatization, rejection, verbal abuse, and blame [17] However, it is important to note that these psychosocial impacts are not unique to LMICs, as literature has documented similar experiences in HICs. Further, the lack of capital due to the delayed employment as a result of the pain and grief caused to the family results in prolonged psychological stress [5].

**Figure 1:**
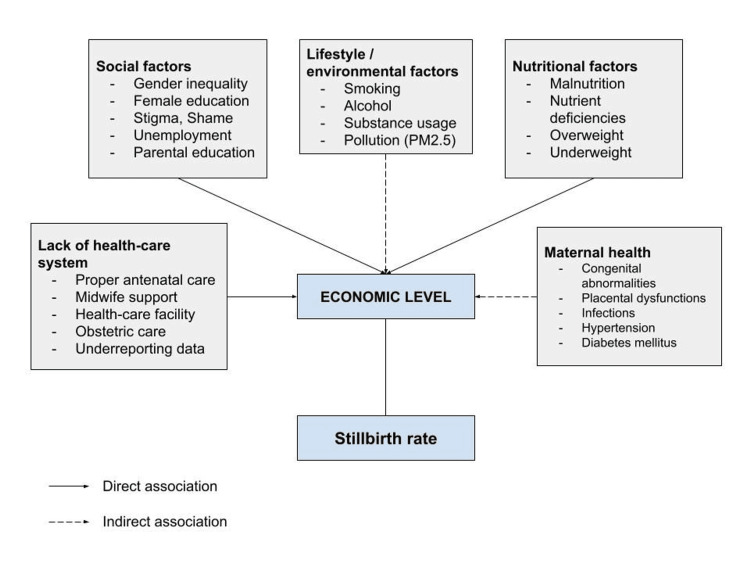
Conceptual framework on various factors of stillbirth in association with the economic level

The conceptual model in Figure 1 depicts the current parameters that increase the burden of stillbirth. This model highlights the multifactorial contributors to stillbirth, with the economic level playing a central mediating role. These interconnected factors emphasize the need for comprehensive strategies to reduce stillbirth rates by addressing socioeconomic, healthcare, and environmental determinants, and this model can guide policymakers and healthcare providers in designing targeted interventions to mitigate the risk of stillbirth.

Government strategies in addressing stillbirth

Government strategies addressing stillbirth have evolved through several significant plans, each with distinct components, implementations, strengths, weaknesses, and barriers. Initially, the Every Newborn Action Plan (ENAP) was formed in 2014 with 194 member states, aiming to reduce neonatal mortality, maternal mortality, and stillbirths globally by 2020 through a comprehensive framework that includes health system strengthening, community engagement, and data-driven interventions. This was not included in the Sustainable Development Goals. Later, ENAP set the target to reduce the stillbirth rates to 12 by 2030 and less than 10 by 2035 for all countries. In 2020, it was estimated that only 32% of countries experienced a reduction in stillbirth rate, while 60% of the countries were not even on target to achieve the ENAP goal; thus, many countries remained off-target [18].

The broad international commitment enhanced resource mobilization and knowledge sharing among countries. In the wake of ENAP, the India Newborn Action Plan (INAP) was launched in 2014 as a committed response to meeting the single-digit Neonatal Mortality Rate goal by 2030. It integrated with India’s Reproductive, Maternal, Newborn, Child, and Adolescent Health Strategy (RMNCH+A) to enhance healthcare delivery. The INAP included a sixth major intervention: pre-conception care, antenatal care, care during labor and childbirth, immediate newborn care, care for healthy newborns, and care beyond survival to achieve this goal. By integrating multiple levels of care and focusing on the continuum from preconception to postpartum, INAP addresses various determinants of health effectively. As of 2021, the stillbirth incidence in India is 13.9 per 1,000 births [19]. Despite its comprehensive framework, challenges such as resource allocation and state-level execution hinder its progress.

Topping the stillbirth chart, South Africa launched the National Health Promotion Policy (NHPP) in 2015 to reduce the stillbirth and maternal mortality rate by providing a continuum of care (CoC) through public health initiatives and community-based interventions. Tailoring interventions to local contexts has shown effectiveness in urban versus rural settings in South Africa. As a result, 8.36% and 2.84% of stillbirths were prevented in urban and rural parts of South Africa, respectively [20]. However, while aiming to reduce disparities, some marginalized communities continue to experience higher stillbirth rates due to systemic barriers.

In 2020, the National Stillbirth Action and Implementation Plan was launched in Australia, prioritizing stillbirth prevention and care, raising awareness, improving stillbirth reporting, ensuring proper data collection, and promoting research with specific timeframes among the Australian population [21]. The emphasis on data collection and research fostered informed decision-making for policy adjustments.

The United Nations International Children’s Emergency Fund (UNICEF) and the World Health Organization (WHO) updated the stillbirth epidemiology report for various countries and regions spanning 2000 to 2021, revealing a global reduction of 35% in the past two decades [22].

Health system limitations, cultural stigma surrounding stillbirth, funding constraints, and lack of coordination among stakeholders pose significant barriers to reducing stillbirth rates in LMICs. Inadequate infrastructure restricts access to quality care, while stigma affects reporting and community engagement. Insufficient financial resources limit the scale of interventions, and poor collaboration among government agencies, NGOs, healthcare providers, and communities often leads to fragmented efforts. The aforementioned government plans and strategies primarily focus on addressing these issues in LMICs rather than HICs. Various plans, strategies, initiatives, and guidelines concerning stillbirth across nations are provided in Table 1.

**Table 1:** Timeline of strategies, plans, initiatives, and guidelines concerning stillbirth LMICs, low- and middle-income countries

Implemented strategies, plans, initiatives, and guidelines	Country	Year	Target/roles	References
Every Newborn Action Plan (ENAP)	Global – WHO	2014	≤12 neonatal deaths per 1,000 live births by 2030; ≤12 stillbirths per 1,000 total births by 2030	[13]
India Newborn Action Plan (INAP)	India	2014	To attain a single-digit stillbirth rate by 2030	[14]
Global Financing Facility in Support of Every Woman Every Child	Global	2015	Supports 36 LMICs; country-driven financing partnership to accelerate efforts to end preventable maternal, newborn, child, and adolescent deaths by 2030	[22]
National Health Promotion Policy (NHPP)	South Africa	2015	To reduce stillbirth and maternal mortality rates in South Africa	[15]
Operational Guidelines for Establishing Sentinel Stillbirth Surveillance System	India	2016	To assist the program managers and health functionaries of sentinel hospitals/health facilities in supporting the establishment of surveillance and guiding the planning and implementation of interventions to reduce stillbirths	[17]
NIHR on Global Health Research Group on Stillbirth Prevention and Management in Sub-Saharan Africa	Sub-Saharan Africa	2017	To prevent and manage stillbirths and neonatal deaths in Sub-Saharan Africa and South Asia	[21]
National Stillbirth Action and Implementation Plan (NSAIP)	Australia	2020	To reduce the number of stillbirths in Australia; to reduce disparities in stillbirth rates between population groups; to raise community awareness and understanding of stillbirth; and to ensure high-quality bereavement care and support are available to families who experience stillbirth	[16]
UNICEF/WHO stillbirth epidemiology report	Global	2020	Global estimates of stillbirth	[1]

Failure in combating stillbirth

Although various strategies and plans have been initiated worldwide, there were 1.9 million stillbirths reported in 2021. As mentioned in the ENAP progress card, among the countries with high stillbirth incidence, only 44% considered stillbirth a priority [18] and implemented plans accordingly. India, Pakistan, Nigeria, the Democratic Republic of the Congo, Ethiopia, and Bangladesh are the top six countries that accounted for half of the stillbirth cases, and these countries share certain socioeconomic characteristics.

In a study conducted in India, stillbirth rates in eight states (Arunachal Pradesh, Goa, Manipur, Meghalaya, Mizoram, Nagaland, Sikkim, and Tripura) were underreported till 2021 [23]. The documentation of stillbirths in India’s data-gathering systems has to be improved for the country to reach its 2030 goal of a single-digit stillbirth rate to track activities to eradicate stillbirths [23]. The National Family Health Survey-4 (NFHS-4) lacks information on modifiable nonmedical risk factors for stillbirth and does not discriminate between stillbirth and infant mortality. Even NFHS-5 offers no commentary on this issue [24].

In Pakistan, the underreporting of stillbirth cases was found to be linked with stigma, lack of care and misunderstanding by healthcare providers, mobility constraints on women, dependency on male members of the family, and lack of obstetrics care [25]. Implementing effective remedies to the problem is complicated by cultural myths about stillbirth and the stigmatization of women. It is crucial to combat social prejudices about stillbirth conditions by improving community knowledge and promoting health literacy regarding the causes of stillbirth and the significance of documentation in countries like Pakistan [25].

A recent study in Afghanistan reported the requirement for immediate financing in their medical system to prevent unrestrained morbidity and fatalities following the dissolution of the Afghan government [26]. Lack of antenatal, peripartum, and postpartum care and general access to medical facilities were found to be major determinants of stillbirth among the sub-Saharan African population [27]; all these determinants were found to be associated with financial crisis and lack of healthcare facilities.

Addressing stillbirth: a multisectoral approach beyond economics

Stillbirth is a multifaceted issue that encompasses various contributing factors, including genetic, environmental, maternal, fetal, and economic influences, and addressing only a single cause will not prevent the issue entirely. Worldwide, LMICs have higher stillbirth rates than HICs; further, compared with any other maternal complication, stillbirth is largely grouped based on the economic levels of countries, stressing the role of capital.

Apart from economic status, the other major risk factors associated with it include maternal factors like fertility, lifestyle, nutrition, and infection; fetal factors like male sex and congenital abnormalities; environmental factors; and genetic defects [28]. Approximately 15-25% of stillbirths are primarily due to genetic causes; it is estimated that 6-17% of fetuses have chromosomal abnormalities. A study on exome sequencing found that missense mutations of COL2A1, PEIZO1, and HNF1B were associated with stillbirth, and ultra-rare variants in PTPN11, SMC3, and FBN2 were known to cause stillbirth [29].

In many underdeveloped nations, advanced maternal age was found to be a substantial risk factor associated with stillbirth. In India, the average age at pregnancy is 35 years, especially in cosmopolitan cities; however, even in rural regions, where the average age at pregnancy is lower (<20 years), stillbirth remains a clinical concern [30].

Numerous studies have identified placental factors, particularly placental abruption, as a major contributor to stillbirth, with estimates ranging from 7.5% to 42% [31]. Insufficient antenatal care during pregnancy increases the risk of stillbirth in LMICs like Tunisia, Peru, Nigeria, Jamaica, and Vietnam [32]. A meta-analysis showed that maternal exposure to PM2.5 and CO increased the risk of stillbirth [33].

Therefore, besides financial distress, the abovementioned maternal, fetal, environmental, and genetic factors pose a major risk in stillbirth. Through proper antenatal interventions, these risks can be reduced, along with screening of high-risk pregnancies, providing proper antenatal care, and ensuring obstetric care.

Future perspectives

Routine antenatal visits will reduce the majority of maternal and fetal complications, and most intrapartum stillbirths are preventable with monitoring and timely intervention [34]. Screening throughout the gestation period is not only restricted for stillbirth prevention but also can be useful in high-risk pregnancies so that these can be detected early for timely intervention [34].

Genetic testing is the key to identifying novel genes and mutations that might contribute to the genetic etiologies of stillbirth, as most people are unaware of these mutations as a cause. The conventional autopsy is a gold standard for determining the cause of fetal death, as it can provide more insights that were inaccessible throughout the pregnancy in 40% of the cases [35]. Cases of stillbirths are often detected in the late gestational stage [36]; therefore, the identification of biomarkers for early detection is extremely important. Omics technology has a major role to play in the detection of biomarkers; however, this field is still in its infancy.

A risk stratification model must be developed in the future, created using large cohorts, to ensure maternal and fetal well-being [37]. To direct public health campaigns and preconception counseling, it is necessary to have more knowledge on preconception and fetal exposure to toxic agents. Community-level awareness by local bodies is the first step to reduce and avoid the stigmatization, taboo, and misconception targeted toward mothers of stillborn infants. Finally, funds and relief should be promptly provided to nations in need to ensure the achievement of a single-digit stillbirth rate by 2030.

## Conclusions

During the past five years, the stillbirth rate has stayed consistent in many regions worldwide. A large difference in stillbirth rate is found between HIC and LMIC. This is mainly due to factors such as maternal health and lifestyle, as well as socioeconomic and geographical factors. Many global and national plans have been implemented to control the number of stillbirths; however, a majority of the countries have failed to achieve this objective. Guidelines concerning stillbirth data collection and registration should be implemented by all countries, especially those that lack a sample surveillance system. However, to attain this successful rate, effective measures for the formulation, implementation, maintenance, and monitoring of the guidelines, policy, and strategies should be established at various levels. However, the most important and immediate need is financial incentive schemes, particularly in LMIC, to ensure the target of single-digit stillbirths is achieved by 2030.
